# Wearable Sensor to Monitor Quality of Upper Limb Task Practice for Stroke Survivors at Home

**DOI:** 10.3390/s24020554

**Published:** 2024-01-16

**Authors:** Na Jin Seo, Kristen Coupland, Christian Finetto, Gabrielle Scronce

**Affiliations:** 1 Department of Rehabilitation Sciences, College of Health Professions, Medical University of South Carolina, Charleston, SC 29425, USA; 2 Department of Health Sciences and Research, College of Health Professions, Medical University of South Carolina, Charleston, SC 29425, USA; coupland@musc.edu (K.C.); finetto@musc.edu (C.F.); scronce@musc.edu (G.S.); 3 Ralph H. Johnson VA Health Care System, Charleston, SC 29401, USA

**Keywords:** stroke, upper extremity, rehabilitation, accelerometer, inertial measurement unit (IMU), wearable sensor, machine learning, remote monitoring, home monitoring

## Abstract

Many stroke survivors experience persistent upper extremity impairment that limits performance in activities of daily living. Upper limb recovery requires high repetitions of task-specific practice. Stroke survivors are often prescribed task practices at home to supplement rehabilitation therapy. A poor quality of task practices, such as the use of compensatory movement patterns, results in maladaptive neuroplasticity and suboptimal motor recovery. There currently lacks a tool for the remote monitoring of movement quality of stroke survivors’ task practices at home. The objective of this study was to evaluate the feasibility of classifying movement quality at home using a wearable IMU. Nineteen stroke survivors wore an IMU sensor on the paretic wrist and performed four functional upper limb tasks in the lab and later at home while videorecording themselves. The lab data served as reference data to classify home movement quality using dynamic time warping. Incorrect and correct movement quality was labeled by a therapist. The home task practice movement quality was classified with an accuracy of 92% and F1 score of 0.95 for all tasks combined. Movement types contributing to misclassification were further investigated. The results support the feasibility of a home movement quality monitoring system to assist with upper limb rehabilitation post stroke.

## 1. Introduction

Stroke is a leading cause of long-term disability worldwide [1]. In the U.S. alone, there are nearly 7 million stroke survivors [2]. Roughly 3% of the adult population have suffered a stroke and related costs are expected to increase to $240 billion in the U.S. by 2030 [3]. Approximately 77% of stroke survivors experience upper extremity motor impairment [4,5,6]. Upper extremity motor impairment decreases individuals’ ability to perform activities of daily living, such as self-care, hygiene, employment, and recreation, thereby diminishing their independence and quality of life [7,8].

Previous research has shown that stroke survivors can recover their upper extremity function through extensive repetitions of task practices [9,10,11,12]. The extensive repetitions of task practices, however, cannot be accomplished within patients’ therapy visits only [13,14,15]. To make up for limited time available within therapy visits, rehabilitation therapists often prescribe a home exercise program comprised of self-directed upper extremity task practices to be performed at home [16]. Those who adhere to the home exercise program to a greater degree have been shown to achieve greater motor recovery, compared to those who do not adhere or who adhere to a lesser degree [17,18]. Thus, the level of adherence to home exercises plays an important role in post-stroke motor recovery [16,17,18,19,20,21]. In addition, if patients do not practice newly learned functional movements at home, their motor capacity gained from therapy visits may not transfer to their activities of daily living, and they may not achieve functional improvement [14,18].

For home exercises to be effective, not only the quantity, but also the quality of task practices is important because the use of compensatory motions for task practices does not address post-stroke motor impairment and may lead to maladaptive plasticity that hinders restoration of motor function [22,23,24]. Thus, many recent studies have aimed to develop systems to monitor the quality of movements for post-stroke rehabilitation, as shown in Table 1. The strength of these previous studies is that they collected data about upper limb movements using an inertial measurement unit (IMU) sensor worn on the wrist for ease of use by patients in the home setting.

The major limitation of these previous studies is that while these systems were developed for home use, the testing of their accuracy was performed in the lab setting only. Validation in the home is warranted to support the use of the monitoring system in the patients’ home. In addition, most studies classified gross movement categories regardless of quality (e.g., functional movements including drinking vs. writing vs. nonfunctional arm movement during walking). One study distinguished compensatory movement patterns from the correct movements in an arm raise task [25]. However, this task was somewhat limited in relevance for activities of daily living typically involving object manipulation using the hand. Another study distinguished the quality of movements for multiple representative functional task practices [26], but only tested healthy adults. Thus, the detection of subtle differences in stroke survivors’ way of performing functional tasks with versus without compensatory movement patterns using a wrist-worn IMU sensor has not been investigated. To this end, the objective of this study was to investigate the feasibility of monitoring stroke survivors’ movement qualities during functional task practices at home using a wrist-worn IMU sensor.

**Table 1:** Summary of previous research classifying upper limb movement types using data from wrist-worn IMU sensors for rehabilitation purposes (RF = random forest, CNN = convolutional neural network, DTW = dynamic time warping, LSTM = Long Short-Term Memory, LDA = linear discriminant analysis, SVM = support vector machine, KNN = K-Nearest Neighbors, CT = classification trees).

Reference	Participant	Method	Setting	Finding
Bochniewicz 2017 [27]	10 healthy persons, 10 stroke survivors	RF	Lab	Classified functional vs. nonfunctional movement
David 2021 [28]	5 healthy persons, 5 hemiparetic patients	Thresholding	Lab	Classified functional vs. nonfunctional movement
Gomez-Arrunategui 2022 [29]	12 stroke survivors	RF, CNN	Lab	Detected reach time and number of reaching gestures during prescribed tasks.
Lee 2018 [25]	9 healthy persons, 11 stroke survivors	RF	Lab	Classified quality of arm raise
Bhagat 2020 [30]	2 persons with spinal cord injury	DTW, LSTM	Lab	Classified cylindrical vs. pincer grasp (to pick up a water bottle vs. pen)
Li 2023 [26]	20 healthy persons	LSTM	Lab	Classified functional task practice quality
Lui 2019 [31]	11 healthy persons	LDA, SVM, KNN, CT	Lab	Classified pre-selected upper limb movements

## 2. Materials and Methods

### 2.1. Participants

Nineteen adult stroke survivors with upper limb hemiparesis participated in the study. Their demographic information is summarized in Table 2. All participants had some hand movement capacity appropriate to engage in the practice of functional tasks involving object manipulation. Participants did not have comorbidities such as orthopedic conditions limiting motion, premorbid neurologic conditions, compromised skin integrity of the wrist, or language barrier or cognitive impairment that precluded following instructions and/or providing consent. Written informed consent was obtained from all participants prior to the study procedure.

### 2.2. Procedure

The study procedure was designed with the intended application of the system as follows. The therapist identifies appropriate therapy goals for a patient during an in-clinic therapy session and prescribes home exercises. Then, a wrist-worn IMU is used to monitor at-home exercise quality, i.e., whether the patient followed the movement pattern learned during the therapy session. Exercise quality is assessed by comparing at-home movements to the in-clinic reference movements using the DTW algorithm.

First, participants came to the laboratory and performed 4 functional tasks using the paretic hand while wearing an IMU sensor (ActiGraph GT9X link, ActiGraph Corporation, Pensacola, FL, USA) on the paretic wrist. An occupational therapist observed their movement quality and identified individual participants’ compensatory movement patterns. Then, they provided feedback to correct their movement patterns, similar with a typical in-clinic therapy session. The 4 functional tasks used in this study are described in Table 3. More details of these tasks along with task instructions and pictures can be found in the study of Li et al., 2023 [26]. These tasks were chosen as representative functional tasks prescribed in home exercise programs from the task practice manual [32] and vary in difficulty. Specifically, tasks 1 and 2 require a cylindrical grasp, with task 1 additionally requiring shoulder flexion. Tasks 3 and 4 are relatively more difficult than tasks 1 and 2, as they require more control and dexterity with the hand. If a participant did not exhibit an incorrect movement pattern for a task, they were instructed to simulate a compensatory movement pattern that they should avoid. Each movement quality was performed with 3 to 6 trials. This number of trials is feasible to accomplish within a standard care rehabilitation therapy session [13]. Each trial’s start and end were time-stamped by the therapist pressing a button on a computer. Trials were annotated for movement quality as correct or incorrect (compensatory or incomplete) by the therapist.

**Figure 1:**
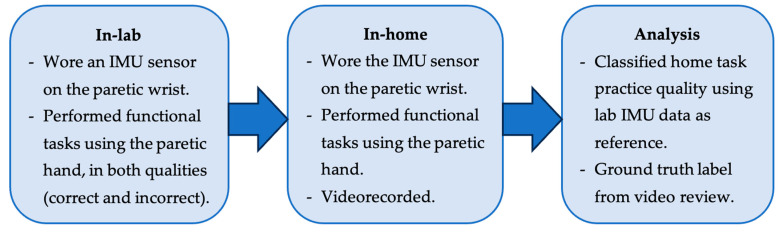
Flow chart of the procedure.

At the end of the in-lab session, participants were instructed to practice these 4 functional tasks at home another day. They were instructed to perform 75 trials of each task with the correct movement pattern learned during the in-lab session. They were provided with an IMU sensor to wear on their paretic wrist at home. The IMU sensor was chosen due to the ease of use by stroke survivors at home, as it requires it wearing an IMU sensor on the wrist just like a watch, thereby increasing the likelihood for feasibility. For ground truth labeling, they also videorecorded themselves performing the functional task practices at home. These videos were later reviewed by the occupational therapist to segment the start and end of each trial and annotate each trial’s movement quality. The occupational therapist had 15 years of experience in treating stroke survivors. The therapist also noted the type for each incorrect movement (e.g., trunk flexion and abnormal forearm supination) to facilitate interpretation of the results. The procedure is summarized in Figure 1.

The IMU sensor recorded acceleration, gyroscope, and magnetometer data in 3 dimensions at 100 Hz both in the lab and home. The IMU sensor data obtained in the lab were used as the reference data for correct vs. incorrect movement patterns. The IMU sensor data obtained in the home were compared against the lab reference data using dynamic time warping (DTW) [33] to classify each home trial’s movement quality.

### 2.3. Analysis

We used participant-specific classification where the participant’s in-lab data served as the references to which their home data were compared. We used this participant-specific approach because individual stroke survivors perform upper limb tasks differently depending on their impairment level, impairment characteristics, and compensatory strategies used. Accordingly, our algorithm of choice was DTW, as DTW enables a patient-specific algorithm for classification. DTW also accommodates varying time durations of movement trials [25,33]. Previous studies using DTW applied to IMU data were able to distinguish cylindrical grasp versus pincer grasp [30], compensatory motions during the paretic arm raise [25], and different exercises [33].

For the lab data, a variety of data processing and DTW methods were explored, and the best method that statistically distinguishes trials of different movement types for a given participant was chosen to be used for analysis of their home data. The rationale for choosing the best method for each participant was that individual participants have different impairment characteristics and compensatory strategies that may be detected better with different methods. Only trials that were clearly determined as correct or incorrect movement, qualified by the therapist, were used for the lab reference data. Exploration of different methods included the use of different combinations of the IMU data, namely the acceleration without gravity, gravity vector, gyroscope, and magnetometer in 3 dimensions. The gravity vector was computed by estimating the sensor orientation from the accelerometer and gyroscope data using a six-axis Kalman filter (imufilter function in MATLAB, MathWorks, Natick, MA, USA). The gravity vector was subtracted from the acceleration data to obtain acceleration without gravity. Exploration of different methods also included data normalization (by the standard deviation for each axis) or lack thereof, as well as various maximum warping sizes for DTW (for MATLAB dtw function’s maxsamp variable ranging from half to 4 times the sampling frequency). To account for different data lengths resulting from DTW, the warped data were projected to the original test data and the DTW distance was computed with the original test data length with a consistent sampling rate.

For each method, DTW distances were computed among all trials of one movement type, and they were also computed between trials of two different movement types of different movement qualities (correct vs. incorrect). The *p*-values for comparison between the two clusters of DTW distances (within the same movement type vs. between two different movement types of different movement qualities) were obtained for all movement types. The *p*-values were obtained using a 1-sided *t*-test with the alternative hypothesis that the DTW distance within the same movement type is smaller than that between two different movement types. The maximum *p*-value was used to indicate the statistical discernability of the movement quality for a given DTW method for a given participant. The method with the smallest *p*-value or *p*-value < 0.05 was chosen.

If multiple methods were able to statistically distinguish trials of different descriptions, then the accuracy of classifying each lab trial in reference to all other lab data was examined using a leave-one-trial-out approach for each method. The method that resulted in the highest accuracy was chosen. If multiple methods performed well for both statistical discernability and lab accuracy, then the method including acceleration without gravity, gravity vector, and gyroscope data with normalization with a longer warping size was chosen. The magnetometer was not considered to provide valuable information about the movement patterns as it relates to the orientation relative to the earth which can change between the lab and home.

Once the best data processing and DTW method was chosen from the lab data for a given participant, the method was applied to analyze that participant’s home data. For each home trial, DTW distances against each of the lab trials were computed. The movement quality label for the lab trial with the minimum DTW distance was used to classify the movement quality of the home trial.

The confusion matrices, along with accuracy and the F1 score, were examined for the classification results. Classification results were examined for all tasks combined as well as for each task. Classification results for individual movement types were also examined. To examine the necessity of collecting gyroscope data in addition to acceleration data at the expense of shorter battery life, the classification accuracy utilizing all available data, including gyroscope, was compared against that using only acceleration data.

## 3. Results

**Figure 2:**
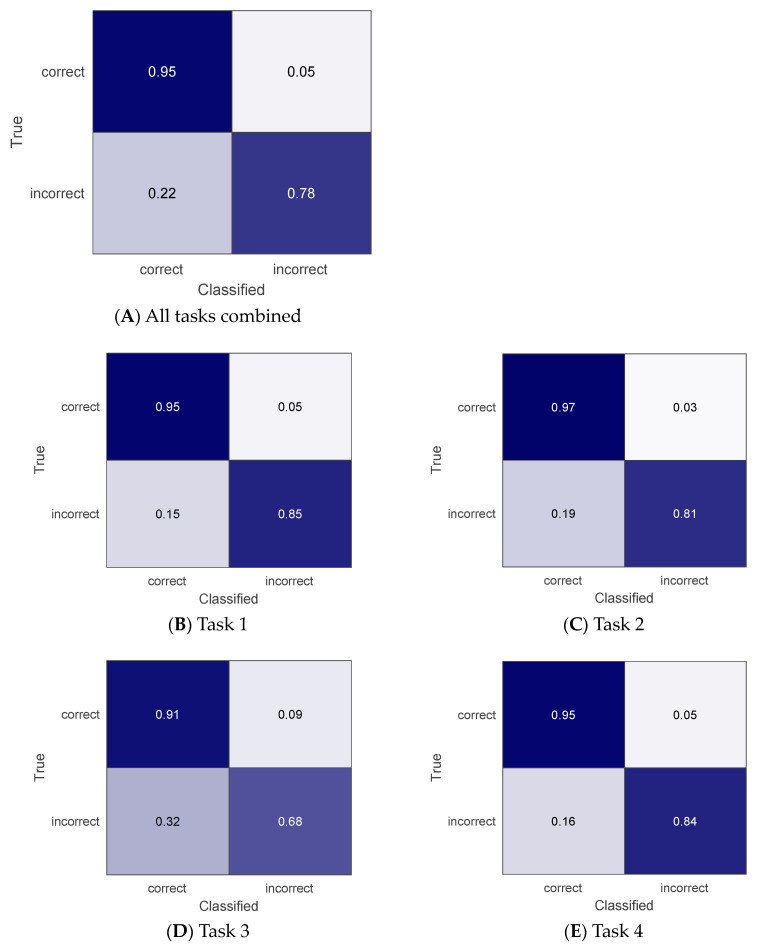
Classification results of the correct vs. incorrect movements at home for all tasks (**A**) and each of the four tasks (**B**–**E**) using all available data including gyroscope. The darker cell color indicates higher percent accuracy.

The results of the movement quality classification for home task practices based on in-lab reference data are shown in confusion matrices in Figure 2. Specifically, Figure 2A shows the results for all tasks and participants’ data combined. The overall accuracy was 92%. The overall F1 score was 0.95. The results for each task are shown in Figure 2B–E. The accuracy was 94%, 95%, 86%, and 93% for task 1 through 4. The F1 score was 0.96, 0.97, 0.91, and 0.96 for task 1 through 4. Across participants, accuracy varied. For task 1, the accuracy ranged from 80% to 100% for individual participants, with a median of 95%. For task 2, the accuracy ranged from 86% to 100% for individual participants, with a median of 97%. For task 3, the range was 68% to 100%, with a median of 86%. For task 4, it ranged from 72% to 100%, with a median of 97%.

**Figure 3:**
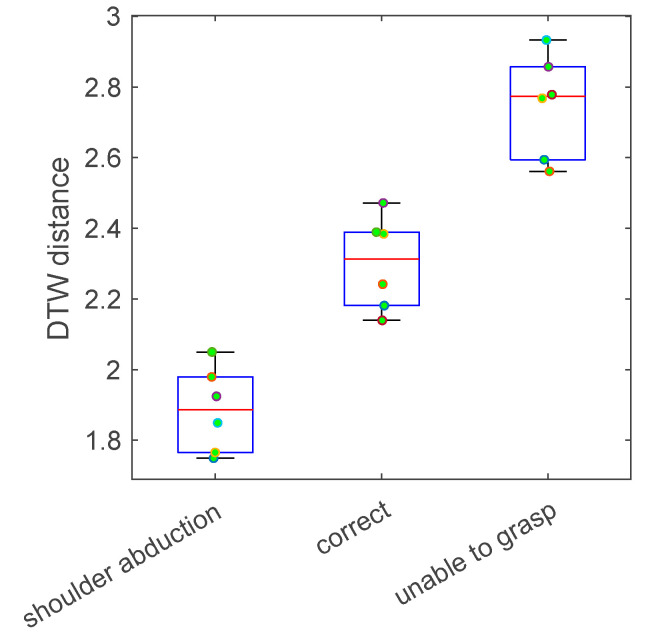
A box plot showing an example DTW distance distribution for a single home task practice trial against lab reference data with 3 movement types (shoulder abduction, correct movement, and unable to grasp the cup) for task 2. Each dot represents the DTW distance between the single home trial and one lab trial. The home trial was performed with shoulder abduction. The DTW distance was smaller between the home trial and the lab trials performed with shoulder abduction. The DTW distance was larger against the lab trials performed correctly and against the lab trials in which the participant was unable to grasp the cup.

An example of the DTW distance distribution for a single home trial against lab reference data is shown in Figure 3 for one participant performing task 2. Specifically, this single home trial was performed with shoulder abduction, according to the video review by the therapist. The DTW distance was computed for this home trial against 3 movement types of in-lab data, which were shoulder abduction, correct movement, and unable to grasp the cup, with 6 trials each. The DTW distance distribution shows that this single home trial had smaller DTW distances to lab trials with shoulder abduction, compared to lab trials with the correct movement pattern or lab trials in which the participant was unable to grasp the cup.

**Figure 4:**
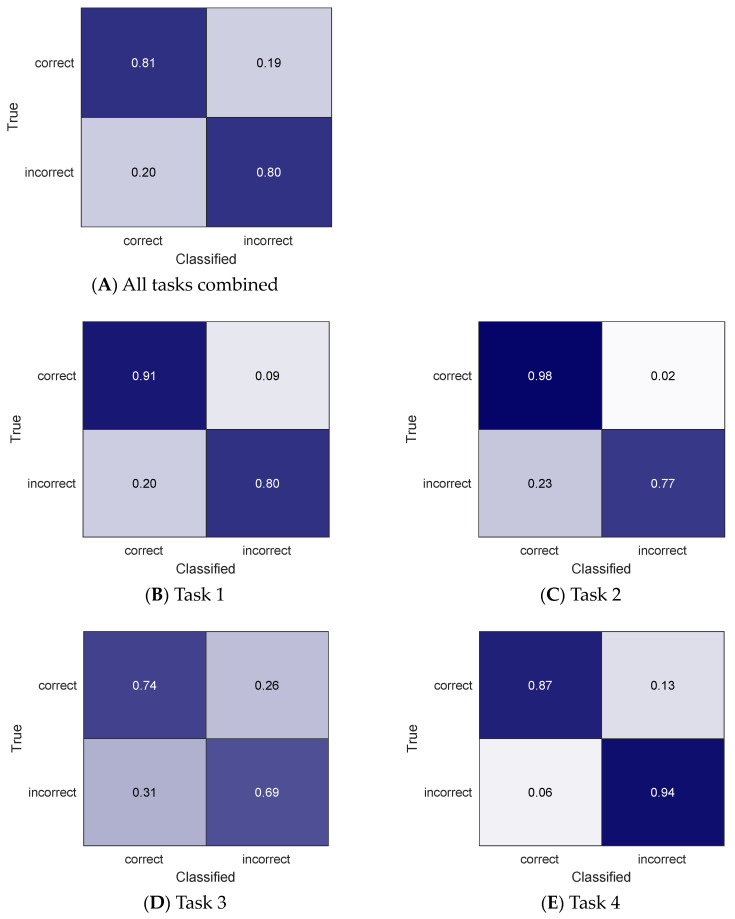
Classification results of the correct vs. incorrect movements at home for all tasks (**A**) and each of the four tasks (**B**–**E**) using only acceleration data. The darker cell color indicates higher percent accuracy.

The results of the movement quality classification for home task practices using only acceleration data (without using gyroscope data) are shown in confusion matrices in Figure 4. For all tasks combined, the accuracy was 81% and the F1 score was 0.88. The accuracy for individual tasks was 89%, 95%, 73%, and 88% for task 1 through 4.

Classification results for individual movement types were examined further to understand the trend (Table 4). Among the incorrect movements, the most frequently observed movement type at home was compensatory movements using the trunk and/or shoulder (e.g., trunk flexion for reaching, and shoulder hike or shoulder abduction for lifting). This movement type accounted for approximately half of the incorrect movements observed at home. This movement type was classified correctly (as incorrect movement), with sensitivity of 86%.

The second most frequently observed incorrect movement type was being unable to complete the task (e.g., unable to grasp, drops before reaching the destination), which accounted for approximately 23% of incorrect movements observed at home. This movement type was classified with sensitivity of 68%. Classification errors were primarily from tasks 3–4, when the block fell while the participant was moving the paretic hand toward the destination. Repeated attempts to grasp without success also contributed to misclassification for tasks 3–4. To a lesser extent, classification errors were from task 1 when the cup fell off the shelf while the participant was placing the cup and/or returning the paretic hand to the table.

Another observed incorrect movement type was use of the nonparetic hand to assist with the task. This movement type of the nonparetic hand use accounted for approximately 13% of the incorrect movements observed at home. The majority occurred in task 3, likely because task 3 was the most difficult of the 4 tasks. This movement type was classified with sensitivity of 62%. Interestingly, use of the nonparetic hand was observed for only three participants in the lab. At home, however, five additional participants used the nonparetic hand. Since these five participants did not display this movement type (use of the nonparetic hand) in the lab, thus lacking this movement type in the reference data, it is explicable that their home data using this movement type could not be classified with high accuracy.

One last observed incorrect movement type observed at home was compensatory grip (e.g., key grip or whole hand grip instead of precision grip for task 4, noncylindrical grip for task 1). This movement type accounted for approximately 13% of the incorrect movements observed at home. This movement type was classified correctly (as incorrect movement), with a sensitivity of 83%. Classification errors were primarily from when the participant dragged the tongs or block in task 3.

## 4. Discussion

This study examined if DTW modeling using data collected via a wrist-worn IMU sensor can be used to discern qualities of upper limb movements during functional task practices at home for stroke survivors. Specifically, person-specific modeling was employed to account for heterogeneous movement patterns across stroke survivors and to focus on individual stroke survivors’ compensatory movement patterns to detect. The individual participants’ data obtained in the lab served as a reference to classify their home data. Ground truth labeling for home data was obtained from video reviews. Overall, the movement quality of stroke survivors’ task practices at home was classified with 92% accuracy. Correct movements at home were classified with 95% sensitivity, while incorrect movements at home were classified with 78% sensitivity. To our best knowledge, this study is the first study to evaluate the performance of movement quality monitoring for stroke survivors *at home*.

The classification results are comparable to those observed in the laboratory in previous studies. Specifically, a previous study found an F1 score of 0.84 when detecting compensatory movement during a simple task of arm raise in neurotypical adults and stroke survivors using the random forest [25]. Another study found an F1 score of 0.89 when detecting compensatory and incomplete movements during functional tasks in neurotypical adults using long short-term memory [26]. The present study found an F1 score of 0.95 in stroke survivors, possibly due to a person-specific modeling approach used compared to other studies.

The classification results are deemed suitable for their intended clinical purpose, considering that they used data from only 1 IMU sensor worn on the paretic wrist and DTW approach with only a limited number of trials of the movement quality from each participant in the lab as the reference data. Compensation from the trunk and shoulder were relatively well classified, as well as compensatory grip strategies such as use of key grip or whole hand grip instead of precision grip for task 4. The trunk and shoulder posture affects the forearm orientation and thus may have affected movement of the wrist, allowing involvement of trunk/shoulder compensation to be detected. The grip posture was also discernable for task 4, possibly because precision grip involves slowing down the hand and wrist extension/stabilization to adequately position the fingertips around the block, while key grip or whole hand grip typically involves greater acceleration without fine adjustment of the hand position. In summary, the findings of this study show that these subtle differences in stroke survivors’ performance of functional tasks with versus without compensatory movement patterns were detectable using a wrist worn IMU sensor and DTW approach.

The lower sensitivity for classification of incorrect movements appears to be due to novel movement types at home that were not used in the lab, including the use of the nonparetic hand to assist, undetected object drops, dragging of the object, and repeated grip attempts not discernable from completion of the task involving gripping tongs, gripping a block, and releasing the block and then tongs for task 3–4. These shortcomings may be addressed as follows. Use of the nonparetic hand to assist with completion of the task may be detected by use of another IMU sensor on the nonparetic wrist. Dropping of the object after being released by the paretic hand can be detected by use of another sensor on the object to detect its movement. Design of the tasks for practice may incorporate different heights for the start and end position (e.g., high and low for grasping and release, respectively) to prevent the possibility of dragging. Use of distinct start and end positions may also introduce a vertical movement between grip and release movements and may help to better distinguish repeated grip attempts from task completions.

In addition, use of novel movement types at home that were unobserved in the lab may be addressed by gleaning from a larger dataset to infer the movement quality. For example, Li et al. (2023) [26] obtained a large dataset encompassing a variety of incorrect movement types from multiple persons with multiple repetitions to develop a deep learning model to classify movement quality. Although this study used data from people without stroke mimicking hemiparetic upper limb movement, the similar approach may be considered for a large number of stroke survivors with various impairment levels and characteristics. A model developed from this large dataset may be fine-tuned using a small amount of data from individual patients via transfer learning [34].

Use of all available data including gyroscope data resulted in better classification results compared to use of only acceleration data. This finding is consistent with previous literature [35]. Collection of only acceleration data could occur for 24 h per day for weeks after charging the battery of the sensor, while collection of all data including gyroscope exhausts the battery only a day or two after charging. Therefore, use of all IMU data to increase classification accuracy would mean that IMU data collection would need to be limited to the home task practice times only and not extended for daily living activities throughout the day, to avoid the need for patients to charge the sensor’s battery at home. Such a trade-off may be lifted in the future with continued enhancement of battery life.

The significant contribution of this study is that this study supports the technical feasibility of monitoring movement qualities of task practices by stroke survivors *at home*. Use of only 1 wrist worn IMU sensor is simple and affords feasibility for long-term home use, as has been demonstrated in previous studies [18,36]. In addition, only a few trials of task practices in the lab were obtained as reference data in order to classify larger home data. The number of trials needed in the lab is well within the feasible number of trials observed in a standard care rehabilitation therapy session [13] and, therefore, maximizes potential for translation to clinical settings. The classification accuracy with the wrist worn IMU sensor in this study was comparable or better compared to that with a camera-based sensor and motion detection algorithm [37]. The DTW algorithm is simple enough to be implemented in a smartphone application for home use without server connection. This simplicity adds to the usability and complements the feasibility toward potential clinical implementation [38].

The anticipated impact of this work is the development of an objective quality monitoring system for home task practices for stroke survivors. Currently, stroke survivors’ adherence to home exercises is typically assessed via self-reports [19,39], which are known to be inaccurate [19,20]. Objective quality monitoring is important because effective home exercises require performance of tasks in the correct movement pattern, without compensatory strategies, to facilitate neuroplasticity and restore motor function post stroke [22,23,24]. While the link between the level of adherence to home exercises in *quantity* and motor recovery has been investigated [17,18], the link between the home exercise *quality* and motor recovery has not been investigated, due to the current lack of such an objective monitoring capability. The objective home task practice quality monitoring system may be incorporated into transfer packages within rehabilitation intervention trials [40,41,42] to increase the real-world use of the paretic hand in stroke survivors. Therefore, this study paves the way for future novel research to investigate the clinical utility of a home task practice quality monitoring system to improve stroke survivors’ motor recovery.

If effective, the objective quality monitoring system may be implemented in clinical practice to improve motor recovery. We envision the integration of this technology in clinical practices and rehabilitation protocols in the following way. The objective monitoring system may assess the quality of task practices at home, provide patients with feedback to improve movement quality for home task practices, and provide a clinician with a summary report to be reviewed as part of remote monitoring [43]. During the in-person therapy appointment, the summary report may provide an opportunity for therapists and stroke survivors to increase the use of correct movement patterns via demonstration, visualization, and instruments [44] and to have conversations about the importance of high-quality home task practices for recovery. A high rate of high-quality home practices could serve as a goal for patients to achieve. A high rate of poor-quality movements may suggest to the clinician that the prescribed task may be too difficult for the patient’s abilities and that an adjustment of task difficulty may be needed to achieve an optimal challenge level to ensure patient engagement for adherence and recovery [32,45]. The monitoring system with a wrist-worn device and smartphone application may be integrated with other rehabilitation technologies such as activity tracking [46], remind-to-move [47,48], sensory stimulation as adjuvant to motor rehabilitation [18,36,49], and home exercise tracking [50] to further the effects. Ultimately, these efforts are expected to enable efficient rehabilitation service for maximal motor recovery post stroke.

Limitations exist in the present study, and future studies may aim to address these limitations. In the present study, if a participant did not exhibit an incorrect movement pattern for a task, they were instructed to simulate a compensatory movement pattern that they should avoid. Considering the patient-specific DTW algorithm, future studies may explore using patient-specific tasks tailored for individual patients’ motor skill level, while examining accuracy, for practical implementation purposes. In addition, only four functional tasks were examined in this study. This limited range of tasks may not fully represent the diverse range of activities needed for comprehensive stroke rehabilitation. Future studies may investigate a broader spectrum of tasks to enhance the applicability and relevance. Likewise, future studies may include a broader spectrum of patients to examine the generalizability of the technical feasibility and usability across stroke survivors with severity levels and impairment characteristics. Such investigation will enhance the relevance to a broader stroke survivor population. Furthermore, only one wrist-worn IMU sensor was used. The use of additional sensors such as on the nonparetic wrist or the object could be explored to enhance accuracy, as discussed above.

In the present study, home task repetitions were segmented via video reviews. Future studies may use a custom smartphone application for stroke survivors to indicate the start and end of each task repetition at home to minimize the burden on stroke survivors to manually count their repetitions and the burden on researchers for video reviews. Such a system including the custom smartphone application and sensors should be investigated for technology acceptance and usability by both stroke survivors [36] and clinicians [51], as insights into the practical challenges, user friendliness, and acceptance in a real-world setting are crucial for successful clinical implementation [38]. While the present study focused on the technical feasibility and immediate accuracy of the system, it does not provide data on long-term clinical outcomes. Future studies may investigate the clinical utility of a home task practice quality monitoring system in improving motor function and quality of life for stroke survivors.

## 5. Conclusions

In summary, the present study provides feasibility for objectively monitoring home task practice movement quality for stroke survivors using a wrist-worn IMU sensor and DTW algorithm. We used only a small amount of movement data obtained from individual participants in the laboratory as the reference data to classify the individuals’ task practices at home on their own. To our best knowledge, this is the first investigation of this kind of movement quality monitoring in stroke survivors at home. The findings of the present study encourage future research to examine the direct linkage between home task practice quality and motor recovery in stroke survivors, as well as personalized feedback to enhance the quality of task practices to improve motor recovery in stroke survivors. Clinical implementation of an objective movement quality monitoring system is expected to provide a means for remote monitoring and increase the efficiency of rehabilitation therapy service for better patient outcomes.

## Figures and Tables

**Table 2:** Participant characteristics.

Characteristics	Descriptive Statistics
Age (mean ± SD years)	61 ± 12
Sex (M/F)	12/7
Time since stroke (mean ± SD years)	4 ± 3
Stroke type (ischemic/hemorrhagic)	14/5
Fugl-Meyer Assessment of Motor Recovery after Stroke—Upper Extremity (mean ± SD out of 66)	45 ± 9

**Table 3:** Description of the four functional tasks.

Task	Description
1. Cup to shelf	Reach to grasp a cup on a table in front of the body, lift the cup to a shelf on top of the table, release the cup on the shelf, and bring the hand back to the table.
2. Cup to mouth	Reach to grasp a cup on the table in front of the body, bring the cup to the mouth, and return the cup to the table, simulating a drinking motion.
3. Tongs use	Reach to grasp tongs on the table, use the tongs to grasp a block and move the block to a destination on the other side of the table crossing the body, and release the tongs back on the table.
4. Finger food	Reach to grasp a block on the table using the pincer or 3-jaw chuck grasp, move the block to a destination away from the body, and bring the hand back to the table.

**Table 4:** Incomplete movement types observed from home, their classification sensitivity, and associated observations.

Movement Type	Sensitivity	Observations
Compensatory trunk and/or shoulder involvement	86%	Trunk flexion for reaching and shoulder hike or shoulder abduction for lifting were identified.
Unable to complete	68%	Classification errors were from earlier object drop and repeated grip attempts in tasks 3–4 and object drop after release in task 1.
Use of the nonparetic hand to assist	62%	Classification errors were from the participants who exhibited this movement type at home but not in the lab. This movement type occurred most for task 3, the most difficult task.
Compensatory grip	83%	Use of key grip or whole hand grip instead of precision grip for task 4 and noncylindrical grip for task 1 were identified. Classification errors were from dragging of the object for task 3 misclassified as correct.

## Data Availability

Data will be shared upon request.
